# From genetics to bariatric surgery and soda taxes: Using all the tools to curb the rising tide of obesity

**DOI:** 10.1371/journal.pmed.1003317

**Published:** 2020-07-31

**Authors:** Adya Misra, Sanjay Basu

**Affiliations:** 1 Public Library of Science, San Francisco, California, United States of America and Cambridge, United Kingdom; 2 Center for Primary Care, Harvard Medical School, Boston, Massachusetts, United States of America

The global prevalence of obesity has virtually tripled since 1975, according to WHO: a 2016 study reported that the number of women living with obesity had increased from 69 million to 390 million, whereas the number of men living with obesity had grown from 31 million to 281 million [1]. Recent estimates indicate that a higher proportion of school-age children are now affected by obesity as compared with 40 years ago, prompting a concerted worldwide effort to reverse this epidemic by 2025 [2]. Often considered to be a consequence of poor lifestyle choices, unhealthy eating behaviours and lack of willpower, obesity has only recently been designated as a chronic disorder, resulting from a complex interplay of genetics and environment [3].

The adverse effects of increased body mass index (BMI), a measure of nutritional status using weight and height, have been documented by a plethora of large observational studies. Large datasets, containing rich genetic and behavioural information about populations, such as the UK Biobank [4], allow researchers to investigate which individuals are at higher risk of obesity. An increased risk of cardiovascular disease, type 2 diabetes (T2D), some cancers and overall mortality due to obesity has been increasingly documented, more recently also in lower-middle-income countries that face a paradoxical burden of malnutrition and obesity. Efforts have been increasingly devoted to understanding the consequences of obesity on mental health and highlight a potential bi-directional relationship—individuals with mental illness might be more at risk of developing obesity, and those with obesity experience weight-related stigma and depression.

In the quest to facilitate changes in policy that can help in tackling the current rising tide of obesity worldwide, this *PLOS Medicine* Special Issue is devoted to the determinants, consequences, prevention and treatment of obesity. The articles included in this issue cover a broad range of topics, reflecting not only the multifaceted role of genetics and the environment in development of obesity, but indeed the severe adverse effects of obesity on physical and mental wellbeing. We discuss findings from some of the articles to portray these diverse themes.

## Determinants of obesity

While environmental and policy interventions to tackle obesity are often discussed separately from metabolic and genetic risk factors, researchers have increasingly linked environmental and biological components of risk to help personalize and direct interventions. The influence of genetic risk for obesity on food choice behaviours is not well understood, although prior work has shown that genetic variants underlying BMI are associated with self-reported eating behaviours, such as increased appetite and uncontrolled eating. The new study from Marie-France Hivert and co-workers utilized objective measures of food choices (including types, amount, and timing of food purchase) over 3 months from hospital employees enrolled in a workplace health promotion randomized trial, and associated these with four previously-identified BMI-associated genetic risk scores [5]. The investigators found that participants in the highest quartile of a genome-wide BMI risk score were more likely to purchase unhealthy food and larger quantities of food, as well as purchase breakfast at later times in the day. Other risk subtypes were associated with skipping meals and not preparing meals at home. The results may inform interventions tailored to changing individuals’ dietary habits.

The study of monogenic forms of obesity offers the opportunity to unravel the role of genetic factors that cause severe disease in some individuals where the environment plays little or no role, offering the hope of targeted therapies. The melanocortin 4 receptor (*MC4R*) is one such gene, mutations in which cause a rare form of early onset obesity. Individuals with obesity who carry pathogenic mutations that cause *MC4R* deficiency present with hyperphagia, increased height, increased fat and lean mass and greater bone mineral density compared to those with obesity and a functional *MC4R*. A recent investigation of *MC4R* mutation carriers revealed that 20% of individuals maintained normal weight, which prompted Nathalie Chami and colleagues to investigate the penetrance of pathogenic mutations in the *MC4R* gene [6]. Analysing data from 450,000 individuals in the UK Biobank, the authors sought to determine the consequence of 59 *MC4R* mutations on body composition and BMI. For 11 of these mutations, carriers had 3.5-fold higher odds of developing obesity than non-carriers.

The authors reported that 15% of *MC4R* mutation carriers were of normal weight, hinting at protective mechanisms that may be offsetting the obesogenic effects of *MC4R* deficiency. In some of these individuals, maintenance of normal weight was attributed to substantially lower polygenic susceptibility to high BMI. The authors also reported that *MC4R* mutation carriers with a low polygenic risk were leaner than non-carriers with a high polygenic risk, suggesting a combined effect of *MC4R* mutations and polygenic susceptibility in body composition. An increased understanding of the relationship between monogenic and polygenic origins of obesity is crucial if we wish to fulfil the vision of providing precision medicine in obesity.

## Consequences of obesity

One of the most extensively studied consequences of high BMI or obesity is T2D. Despite this well-established link, there is considerable individual variation in the progression of T2D—as some patients are able to maintain glycaemic control while others experience deterioration in clinical symptoms. Using routinely collected data in Hong Kong, Guozhi Jiang *et al*. report that rapid glycaemic progression was associated with patients who were diagnosed at a young age, had high HbA1c, high triglycerides, used tobacco or had either very low or very high BMI [7]. Apart from reversible lifestyle factors, the authors sought to understand whether any genetic factors influence this rapid deterioration observed in some patients with T2D. Notably, a polygenic risk score including variants related to T2D, but not BMI, was found to be associated with glycaemic progression. This study highlights the importance of using both genetic and lifestyle indicators for risk stratification in patients.

There are other health related consequences of high BMI, such as cardiovascular disease, chronic kidney disease, musculoskeletal disorders, certain cancers and mortality. Using the modelling framework in the Global Burden of Disease study 2017, Haijiang Dai and colleagues report that 2.4 million deaths were attributed to high BMI in 2017 and that the number of years of life lost to ill-health due to high BMI had more than doubled since 1990 [8]. The authors also found that cardiovascular disease was the largest contributor to years lost due to high BMI-related ill-health. Interestingly, these analyses show that females over the age of 70 years and males younger than 70 years were at high risk of death and years lost to ill-health due to high BMI, suggesting that obesity prevention programs should be adapted to reach these high risk populations. Worryingly, there was an increasing trend in high BMI-related disease burden in countries with a lower sociodemographic index. While the reasons for this might be multifaceted, this double burden of undernutrition and obesity is likely to add pressure to resource-limited health systems.

## Prevention of obesity

Among the major concerns for research into obesity control is how fiscal policies to reduce sugar-sweetened beverage (SSB) consumption—particularly taxes on SSBs—would be expected to impact upon different demographic groups in lower-middle-income countries facing the dual burdens of poverty and obesity-related disease. In their article on projecting SSB tax impacts in Argentina, Joanne Penko *et al*. used a well-established simulation model that projects 30 cardiovascular and mortality events to identify differential demographic and morbidity impacts [9]. They modelled scenarios that include a range of potential compensatory behaviours (i.e., increased consumption of other products accompanying decreased consumption of SSBs), which is critical to understanding the net impact of a tax. They estimated that even relatively modest reductions in consumption with sizable compensation would be expected to have meaningful benefits for cardiovascular and metabolic disease outcomes, which is important for addressing policy scepticism in the context of continued political challenges to implementing SSB taxation in Latin America.

Also in the Latin American context, Reyes *et al*. investigate food reformulation policies in Chile [10]. In June 2016, Chile implemented a broad set of regulatory policies intended to decrease obesity, including the use of warning labels on some foods and beverages considered unhealthy, restrictions to children-targeted marketing, and restrictions on the sale of certain foods and beverages in schools and nurseries. Using nutrient content information from over 4,000 packaged foods and beverages available in supermarkets and candy stores in the capital, Santiago, the investigators found that after implementation of these policies, metrics of the nutritional quality of several foods and beverages improved, specifically through declines in sugar and sodium content. The results of this mandatory national policy provide evidence that may be generalizable beyond Chile or Latin America.

## Treatment of obesity

Treatment of obesity depends on the nature and severity of symptoms, with lifestyle modification, nutrition advice and increased physical activity recommended for those with a BMI of 25–30 kg/m^2^. Bariatric surgery in the UK is considered for patients with a BMI of 40kg/m^2^ or more, or if several co-morbidities are present, in order to reduce the risk of obesity-related mortality. Although several clinical trials have demonstrated the effectiveness of bariatric surgery, Tom Wiggins *et al*. report that these findings are not limited to specialised metabolic surgery centres and can be generalised to the wider population [11]. Using pooled data from studies utilising large administrative databases, the authors showed that bariatric surgery is associated with a lower risk of new onset diabetes, hypertension, cardiovascular disease and a reduced risk of mortality. Bariatric surgery offers a powerful therapeutic option to patients who are unable to reduce their weight with other interventions and develop severe comorbidities, however it remains uncertain whether the reduction in mortality risk is comparable to that of the general population.

These and other articles in this Special Issue exhibit the multidimensional nature of obesity research and the complex relationship between the risk factors and consequences of obesity. We received submissions from around the globe, highlighting the context-specific challenges of, and potential solutions to, tackling the obesity epidemic. Developments in genetics, pharmacogenetics and epigenetics offer hope in achieving the promise of precision medicine for obesity. At the population level, those in favour of a “one size fits all” approach may argue that prevention of obesity can perhaps be achieved by using warning labels and taxation of unhealthy food and beverages, for instance. Sugar levies have been implemented in Chile [12], Portugal [13], and the UK [14], but it remains to be seen whether this has encouraged manufacturers to reduce added sugars and avoid the levy and, as a result, whether individuals are consuming fewer sugary beverages. With Latin American countries taking the lead, we anticipate that findings from Mexico, Argentina and Chile published in this Special Issue will encourage policymakers worldwide to adopt similar measures. The inexorable growth of obesity requires a multi-pronged approach involving personal- and population-level preventive measures as well as reliable long-term solutions to stop the progression of disease. Clinicians and policymakers must take into account the individual differences within groups and create personalised treatment plans to ensure optimal treatment outcomes. We hope that research published in this Special Issue will encourage further investigation of the gaps in obesity research and offer sustainable solutions to combat this global epidemic.
